# TRIP-1 Promotes the Assembly of an ECM That Contains Extracellular Vesicles and Factors That Modulate Angiogenesis

**DOI:** 10.3389/fphys.2018.01092

**Published:** 2018-08-15

**Authors:** Yinghua Chen, Anne George

**Affiliations:** Brodie Tooth Development Genetics and Regenerative Medicine Research Laboratory, Department of Oral Biology, University of Illinois at Chicago, Chicago, IL, United States

**Keywords:** TRIP-1, ECM, bone, mineralization, microvesicles, angiogenesis, matrix

## Abstract

Transforming growth factor beta receptor II interacting protein-1 (TRIP-1) was recently localized in the mineralized matrices of bone and dentin. The function of TRIP-1 in the ECM is enigmatic, as it is known to function as an intracellular endoplasmic reticulum protein during protein synthesis. Based on its localization pattern in bones and teeth, we posited that TRIP-1 must function as a regulatory protein with multiple functions during mineralization. In this study, we determined the *in vivo* function of TRIP-1 by an implantation assay performed using recombinant TRIP-1 and TRIP-1 overexpressing and knocked down cells embedded in a 3D biomimetic scaffold. After 4 weeks, the subcutaneous tissues from TRIP-1 overexpressing cells and scaffolds containing recombinant TRIP-1 showed higher expression levels of several ECM proteins such as fibronectin and collagen I. Picrosirius red and polarized microscopy was used to identify the birefringence of the collagen fibrils in the extracellular matrix (ECM). Interestingly, knockdown of TRIP-1 resulted in lower fibronectin and downregulation of the activation of the ERK MAP kinase. We further demonstrate that TRIP-1 overexpression leads to higher expression of pro-angiogenic marker VEGF and downregulation of anti-angiogenic factors such as pigment epithelium-derived factor and thrombospondin. Field emission scanning electron microscope results demonstrated that TRIP-1 overexpressing cells released large amount of extracellular microvesicles which were localized on the fibrillar matrix in the ECM. Overall, this study demonstrates that TRIP-1 can promote secretion of extracellular vesicles, synthesis of key osteogenic ECM matrix proteins and promote angiogenesis.

## Introduction

Osteoblasts and odontoblasts are bone and dentin forming cells that synthesize and assemble a specialized extracellular matrix (Gajjeraman et al., 2007; George and Veis, 2008). Biological apatite deposited in the extracellular matrix (ECM) is orchestrated by an organic extracellular matrix that controls mineral nucleation and growth (He et al., 2003). This extracellular matrix is a complex network of collagens, glycoproteins such as fibronectin, SIBLING protein family members, phosphoproteins, proteoglycans, and polysaccharides (He et al., 2005; Ravindran and George, 2014). During bone development several of these regulatory proteins are expressed at defined stages to perform specific function in maintaining the structure and function of bone (Hao et al., 2004). In hard tissues, the calcified ECM matrix acts as an osteoinductive matrix and influences cell behavior and influences physiological processes such as angiogenesis (Nienhuijs et al., 2011; Ajlan et al., 2015). However, there are yet unidentified proteins in the matrix that may play a regulatory role in initiating physiological processes such as cell differentiation and angiogenesis during the formation of mineralized tissues.

To understand the mechanisms of matrix mineralization, we have identified a potentially new matrix protein, TRIP-1 [transforming growth factor beta (TGF-β) receptor II interacting protein-1] (Ramachandran et al., 2012). Earlier reports show that TRIP-1 is localized in the ER and functions in concert with other subunits of the initiation complex to regulate protein synthesis (Choy and Derynck, 1998; Perez et al., 2011; Herrmannova et al., 2012). Therefore, its localization in the matrix was enigmatic. TRIP-1 expression is regulated spatially and temporally during skeletal development. During intramembranous bone development, predominant localization was seen during mouse development as early as embryo day 20 in preosteoblasts and differentiated osteoblasts. During endochondral ossification process, TRIP-1 expression was observed early during development in the epiphyseal growth plate. Interestingly TRIP-1 was observed in the extracellular matrix of the proliferating chondrocytes (Ramachandran et al., 2012). With the development of the primary spongiosa, TRIP-1 expression shifted from the cartilage and was abundantly expressed in the osteoid of the bony spicules. Within mature bone, TRIP-1 expression persisted in the periosteum, osteoblasts, osteocytes and in the bone matrix. Interestingly, its localization in the primary spongiosa suggests that TRIP-1 might function in the extracellular matrix to regulate mineralization as well as promote angiogenesis. A pivotal role for TRIP-1 in mineralization has been recently demonstrated in an *in vitro* nucleation model (Ramachandran et al., 2016, 2018).

To investigate other potential functions of TRIP-1 in the extracellular matrix, genetically modified cell lines overexpressing (TRIP1-OE) and knocked down TRIP-1 (TRIP1-KD) in preosteoblast MC3T3-E1cells were generated. The morphology of the genetically modified cells, nature of the deposited ECM matrix, osteogenic potential and its influence on neovascularization were studied.

## Materials and Methods

### Expression and Purification of Recombinant TRIP-1

Recombinant TRIP-1 protein was expressed in bacteria using the pQE-30 plasmid (Qiagen) system. Briefly, 973-bp fragment corresponding to the coding region of TRIP-1 was cloned into *Eco*RI/*Xho*I restriction sites in pQE-30 vector. This plasmid was transformed into *E. coli* bacteria BL21-Gold (DE3). The protein expression was induced by the addition of 1 mM IPTG. The expressed protein was purified using Ni-NTA agarose (Qiagen) column under native conditions. The manufacturer’s protocol was followed for protein elution. Specifically, the buffer containing 250 mM imidazole, pH 8.0 was used to elute TRIP-1 from the column.

### Overexpression and Knock-Down of TRIP-1

Stable overexpression was performed by transfecting the pECFP vector (Clontech Laboratories, Mountain View, CA, United States) containing the rat TRIP-1 cDNA in MC3T3-E1 cells as published earlier (Ramachandran et al., 2016). Cells mock transfected with the empty vector served as the control. For knock-down studies, mission shRNA clones for TRIP-1 (Sigma-Aldrich, St. Louis, MO, United States) was used to generate a stable cell-line. Cells transfected with scrambled shRNA was used as control. Real-time PCR analysis was used to confirm the expression levels of TRIP-1 in the genetically modified MC3T3-E1 cells (Ramachandran et al., 2012).

### Isolation of the ECM

MC3T3-TRIP1-OE (overexpression) and MC3T3-TRIP1-KD (knocked-down) cells were grown to confluence on cover glass for ECM isolation. Cells were lysed by incubating for 15 min with 0.5% TritonX-100, 0.01 M sodium phosphate, 0.15 M NaCl, pH 7.4 at 37°C and 5% CO2, followed by incubation for 10 min under the same conditions with 0.25 M ammonium hydroxide. The cover glass were then washed with 0.02 M Tris-HCl, pH 7.4, 0.15 M NaCl and 0.05% Tween-20. A final wash was performed with HBS buffer. The ECM was then fixed in 4% paraformaldehyde and subjected to immunostaining with rabbit anti-DMP1 antibody (1/2000), rabbit anti-GRP-78 (1/500), anti-fibronectin (1/100 Sigma-Aldrich), and rabbit anti-DMP4 antibodies (developed and characterized in our laboratory).

### *In vivo* Assay Using a Subcutaneous Implantation Model in Mice to Determine the Osteogenic and Angiogenic Potential of TRIP-1

Two groups of implantation experiments were conducted in this study. The first group consisted of hydrogel scaffold pretreated with rTRIP-1 and seeded with 2 × 10^6^ MC3T3-E1 cells while the hydrogel containing cells with no treatment served as control. In the second group control and genetically modified cells were seeded at a density of 2 × 10^6^ cells per scaffold. LZ–Control protein at 7% concentration was used to make hydrogel scaffolds. Preparation of the leucine zipper (LZ) hydrogel scaffold was performed as published earlier (Huang et al., 2014; Ravindran et al., 2014). LZ hydrogel scaffold was adsorbed with 100 μg rTRIP-1 protein and LZ hydrogel with no treatment served as control were used. The cell seeded scaffolds were cultured *in vitro* for 48 h and were then implanted subcutaneously on the back of 1 month old athymic nude mice (Charles River Laboratories). Four weeks post implantation, the animals were sacrificed and the scaffolds were retrieved, fixed in 4% neutral buffered formalin, embedded and sectioned into 5 μm thick sections for histological evaluation. All animal experiments were performed as per protocol approved by the UIC animal care committee (Assurance number A-3460-01).

### Histology and Immunohistochemistry

All sections were deparaffinized in xylene, rehydrated in graded ethanol solutions, and hematoxylin and eosin (H&E) staining was performed as per published protocols (Hao et al., 2009). Immunohistochemistry using peroxidase conjugated secondary antibodies or fluorescent probes according to published protocols. The following antibodies were used: Rabbit anti-cluster of differentiation (CD31) antibody (1/100; Abcam), rabbit anti-vascular endothelial growth factor (VEGF) antibody (1/100, Santa Cruz Biotechnology), rabbit anti-pigment epithelium-derived factor (PEDF) antibody (1/500; Millipore), mouse anti-von Willebrand factor (vWF) antibody (1/100; Santa Cruz Biotechnology); rabbit anti fibronectin (FN) antibody (1/100; Sigma-Aldrich), goat alkaline phosphatase (ALP) antibody (1/100; Thermo Fisher Scientific), mouse dentin matrix protein 1 (DMP1) antibody (1/10000; kind gift from Dr. Qin, University of Texas), rabbit type I Collagen (1/100; Abcam) and mouse anti-phospho serine antibody (1/100; Sigma-Aldrich), mouse anti-thrombospondin (1/100; Sigma-Aldrich). For all immunohistochemistry experiments with fluorescent secondary antibody labeling, anti-mouse FITC (1/100; Sigma-Aldrich) and anti-rabbit (TRITC 1/100; Sigma-Aldrich) antibodies were used. All fluorescently stained sections were imaged at the University of Illinois at Chicago Research Resource Center core imaging facility. Imaging was performed using a Zeiss LSM 710 confocal microscope equipped with Zen image analysis software. Peroxidase stained sections were imaged using a Zeiss Axio-observer D1 microscope. All comparative fluorescence images were obtained using the same imaging conditions.

### Western Blot Analysis

Total proteins were isolated as published earlier (Ramachandran et al., 2012). The blots were blocked with 5% nonfat milk, probed with rabbit polyclonal fibronectin antibody (1:2000) and rabbit pERK ½ (1:200; Santa Cruz Biotechnology).

### Picrosirius Red Staining

Paraffin embedded sections were stained using the dye picrosirius red to demonstrate the presence of collagen in the ECM. Birefringence pattern of collagen fibers was determined using Zeiss polarized light microscope.

### Image Processing for Quantification of Collagen Fiber

Representative images from picrosirius red stained sections were processed to obtain collagen fiber metrics at defined regions of interest. Collagen length and diameter were measured using CT-FIRE fiber detection software (LOCI; Madison, WI, United States) (Bredfeldt et al., 2014).

### Scanning Electron Microscopy

MC3T3-E1, MC3T3-E1-TRIP1-OE, and MC3T3-E1-TRIP1-KD cells were grown on 12-mm cover glass placed in 12-well tissue culture plates in osteogenic differentiation media, i.e., normal growth medium supplemented with 10 mM β-glycerophosphate, 100 mg/ml ascorbic acid and 10 nM dexamethasone for 14 days. They were then washed twice with PBS and fixed in 1.5% glutaraldehyde in 0.15 M sodium cacodylate buffer for 24 h at 4°C. After washing twice with PBS, the samples were dehydrated in graded ethanol solutions and then completely dehydrated by immersing them in a solution of hexamethyldisilazane (HMDS) for 10 min followed by air–drying inside a tissue culture hood. The samples were coated with 10 μm thickness of platinum/palladium and imaged on a scanning electron microscope (Hitachi S-3000N). Field emission scanning electron microscope (FESEM) JEOL JSM-6320 was used to image collagen fibrils and vesicles in the matrix.

### Exosome Isolation and TEM

Media was collected from confluent MC3T3-E1-TRIP1-OE cells and centrifuged at 400 *g* for 5 min to remove debris. The cleared supernatants were processed by sequential ultracentrifugation according to published protocols (Xu et al., 2017). The final pellet was suspended in 200 μl of PBS and processed for transmission electron microscopy (TEM).

Twenty microliters of the resuspended exosomes were added to 300 mesh formvar coated nickel grids and viewed without coating. For immunogold cytochemistry, published protocols were followed (Ramachandran et al., 2016). Briefly, the grids were fixed in 10% neutral formalin, permeabilized with PBS containing 1% Trton-X100 for 30 min and blocked with 3% BSA for 1 h at room temperature. The grids were then incubated with rabbit polyclonal CD63 (1/500) and mouse monoclonal TRIP1 (1/500) antibody. The grids were washed followed by incubation with gold conjugated anti-rabbit IgG (20 nm) and anti-mouse IgG (10 nm) washed and analyzed by TEM.

### Statistical Analysis

Data are presented as the mean ± standard deviation of at least three independent experiments. Statistical analysis of the data was calculated using the Student’s *t*-test. *P* < 0.05 was considered significant.

## Results

### Histological Evaluation of the Explant Tissue

**FIGURE 1:**
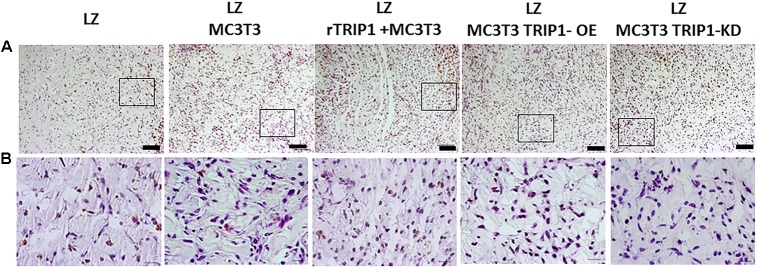
Cellular architecture of *de novo* formed tissue analyzed by histological H&E staining. **(A)** Low power magnification of the tissues from different groups as indicated. Connective tissue stains as light pink and the cell nuclei stains blue. Bar represents 100 microns. **(B)** Higher magnification of the boxed areas from panel **(A)**. Bar represents 20 microns.

Hematoxylin and eosin staining was used to define the cellular and matrix content in the stroma generated by different cell types used in this study. Results in **Figures 1A,B** showed abundant ECM produced in the LZ-rTRIP1 and LZ-TRIP1-OE group. All the four groups showed high cellular content. The control LZ scaffold contained was infiltrated by few host cells.

**FIGURE 2:**
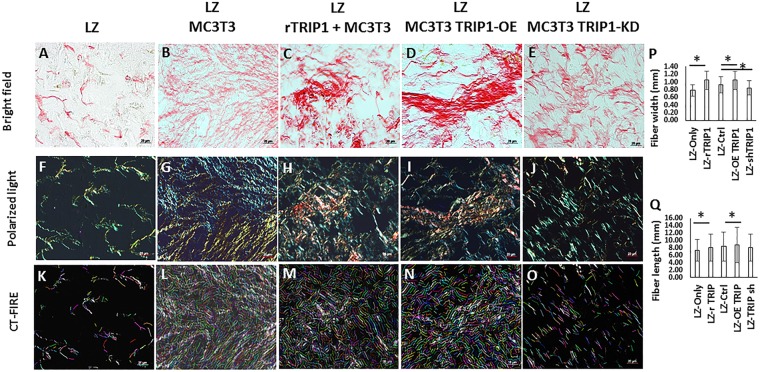
Histology of *de novo* formed tissues stained with picrosirius red to assess the presence of collagen fibrils in the matrix. **(A–E)** Picrosirius red stained tissues obtained from different experimental groups under light microscopy. Bar represents 20 microns. **(F–J)** Picrosirius red stained tissues obtained from different experimental groups under polarized light. Bar represents 20 microns. **(K–O)** CT-FIRE was used to quantify collagen fiber length and diameter in different groups and the quantifications are represented in panels **(P,Q)**. Stastically significant differences are indicated. ^∗^*P* ≤ 0.05.

Picrosirius red staining of the ECM matrix under bright field microscopy showed that the collagen fibrils appeared uniformly dark pink (**Figures 2A–E**) and birefringence colors under polarized light microscopy in all the groups (**Figures 2F–J**). In the groups containing TRIP-1 the collagen fibrils were thick and tightly packed and exhibited yellowish orange birefringent colors (**Figures 2H–I**). In the absence of TRIP-1 loose network of thin fibers, weakly birefringent ranging from greenish blue to yellow were observed (**Figure 2J**). CT-FIRE fiber analysis of images (**Figures 2K–O**) revealed that the collagen fibrils were thicker and longer in groups containing TRIP-1 (**Figures 2P,Q**).

### TRIP-1 Overexpressing MC3T3-E1 Cells Produce a Unique Extracellular Matrix Containing Extracellular Vesicles

**FIGURE 3:**
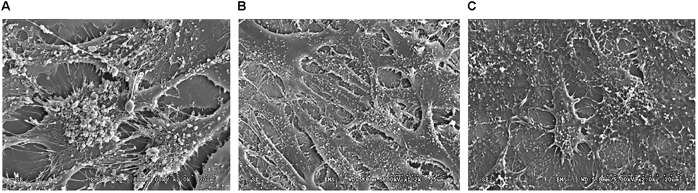
SEM image of the cells and the secreted microvesicles when cultured for 14 days in osteogenic media. The SE micrographs are representative images of MC3T3-E1 cells **(A)**, TRIP1-OE cells **(B)**, and TRIP1-KD cells **(C)** grown in osteogenic media for 14 days. Note the large amount of extracellular vesicles released by TRIP1-OE cells.

**FIGURE 4:**
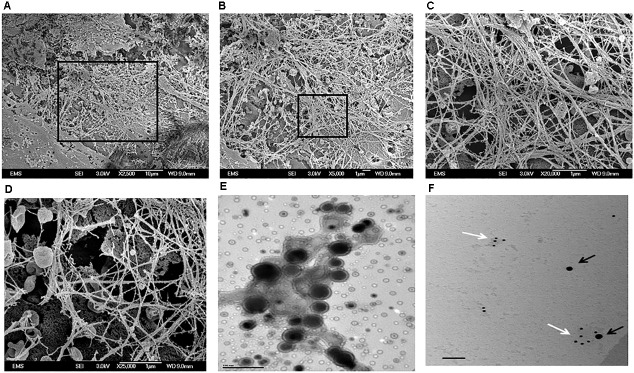
Electron Microscopy images of the secreted ECM by TRIP1 overexpressing cells after 14 days of culture in osteogenic media. Representative low-magnification FESEM images of the secreted ECM from MC3T3-TRIP1-OE cells **(A);** high magnification image of the boxed area in panel **(A)** showing a highly fibrous ECM with embedded extracellular vesicles **(B)**; high magnification from the boxed area in panel **(B)** showing the dense fibrous matrix **(C)**; high magnification image showing the cup-shaped extracellular vesicles localized specifically on the fibrils **(D)**. Magnification as indicated. Representative TEM image of unstained extracellular vesicles showing the morphology of the vesicles isolated from TRIP1-OE cells under mineralization **(E).** Scale bar 100 nm. Representative TEM image of solubilized exosomes showing the presence of immunogold labeled CD-63 (black arrows, 20 nm) and TRIP-1 (white arrows,10 nm) **(F).** Scale bar 100 nm.

Genetically-modified cell-lines established earlier were used in this study to investigate the morphology and evaluate the presence of key matrix components in the ECM when cultured under osteogenic conditions for 14 days. SEM analysis showed numerous extracellular vesicles (EV) on the surface of all the three cell types (**Figure 3**). However, TRIP1-OE cells showed highly elongated cellular processes coated with numerous EV (**Figure 3B**) while, TRIP1-KD cells had remarkably low amounts of EV (**Figure 3C**). The control MC3T3-E1 cells which had endogenous levels of TRIP-1 released more microvesicles than the TRIP1-KD cells (**Figure 3A**). High-resolution visualization of the ECM using Field Emission SEM showed the ultrastructure of the heterogeneous matrix. Images in **Figures 4A,B** showed that TRIP1-OE cells secreted a network of fibrillary ECM and the diameter of the fibrils were highly homogeneous (**Figure 4C**). Adhered to the fibrils were numerous vesicles (**Figure 4D**). TEM analysis of the EV isolated by ultracentrifugation showed nanometer sized heterogenous population of exosome-like particles containing electron-dense particles in the core (**Figure 4E**). Analysis of the solubilized exosomes by immunogold labeling show the presence of CD-63 an exosome marker (**Figure 4F**, black arrows) and also contained TRIP-1 (**Figure 4F**, white arrows).

**FIGURE 5:**
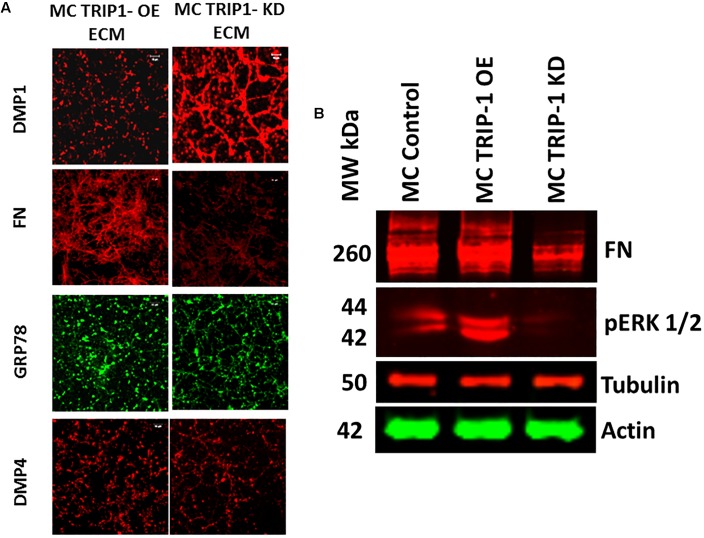
Expression of key extracellular matrix proteins involved in mineralization. Representative confocal micrographs showing the expression of DMP1, FN, GRP-78 and DMP4 in the ECM of MC3T3-TRIP1-OE and MC3T3-TRIP1-KD cells **(A)**. Western blot of total proteins showing the presence of FN, pERK in MC3T3-E1, MC3T3-TRIP1-OE, and MC3T3-TRIP1-KD cells. Note lower expression of FN and pERK1/2 in TRIP1-KD cells. Tubulin and actin were used as loading controls **(B).**

We next isolated the ECM from TRIP1-OE and TRIP1-KD cells and examined for several matrix proteins such as DMP1, FN, GRP78, and DMP4 (Fam20C) by IHC (**Figure 5A**). Interestingly, fibronectin the principal protein of the ECM was present in low amounts in TRIP1-KD cells when compared to TRIP-OE cells. Expression levels of GRP-78 and DMP4 were also modestly reduced, however, higher expression levels of DMP1 was observed with TRIP1-KD cells. Additionally, Western blot analysis in **Figure 5B** confirmed lower expression levels of fibronectin in TRIP1-KD cells. As fibronectin in the matrix is known to activate ERK1/2 MAP kinase, therefore, we sought to determine if TRIP-1 mediated fibronectin expression could regulate cellular differentiation by activating ERK MAP kinase. Western blot results in **Figure 5B** also confirm ERK activation in response to TRIP-1 overexpression and attenuated in TRIP1-KD cells.

### TRIP-1 Overexpression Promotes Cellular Differentiation as Assessed by the Presence of Biomineralization Regulators

**FIGURE 6:**
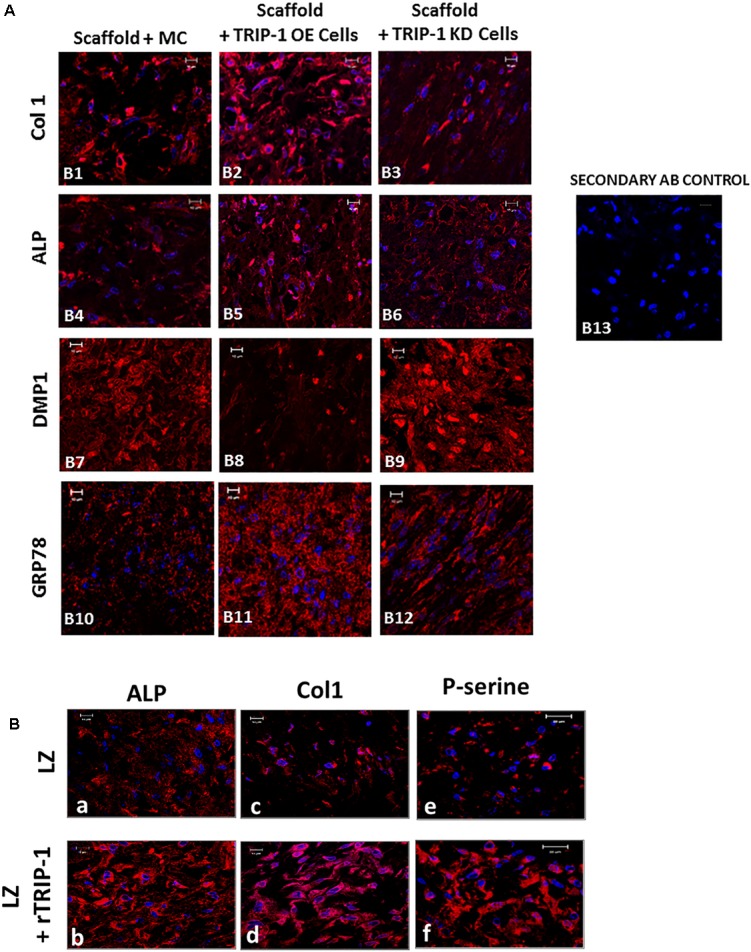
**(A)** Effect of TRIP-1 overexpression and knock-down and its influence on secretion of matrix proteins *in vivo*. Representative confocal micrographs showing the localization of Col1 **(B1–B3)**, ALP **(B4–B6)**, DMP1 **(B7–B9)**, GRP-78 **(B10–B12)** in implanted scaffold containing control MC3T3-E1, TRIP1-OE, and TRIP1-KD cells, respectively. Scale bar represents 20 μm for all images. **(B)** Effect of rTRIP-1 adsorbed on LZ-scaffold containing MC3T3-E1 cells on matrix proteins *in vivo*. Representative confocal micrographs showing the immunohistochemical localization of ALP **(a,b)**, Col1 **(c,d)**, P-serine **(e,f)** in implanted control scaffold and scaffold +rTRIP-1, respectively. Note increased expression levels of osteogenic markers with rTRIP-1 treatment. Scale bar represents 20 μm for all images.

*In vivo* function of TRIP-1 in biomineralization was assessed by examining the explants obtained from subcutaneous implantation of 3D-scaffolds containing MC3T3-E1, TRIP1-OE, or TRIP1-KD cells. Use of LZ hydrogel scaffolds for *in vivo* analysis was established and published (Huang et al., 2014). The explants were harvested after 4 weeks and subjected to immunohistochemical analysis. Results show an increase in the expression of the major ECM proteins (**Figure 6A**) such as type I collagen (Col1) (B1–B3), ALP a key enzyme that initiates mineralization (B4–B6) and Glucose regulated protein 78 (GRP-78) that we have recently identified in the matrices of bone and dentin (B10–B12). However, the expression level of dentin matrix protein 1 (DMP1) a key regulatory matrix protein in bone and dentin mineralization was higher in TRIP1-KD cells (B7–B9). Control rabbit and mouse secondary (B13) did not show any staining.

With the scaffolds containing MC3T3-E1 cells adsorbed with rTRIP1 on the LZ scaffold (**Figure 6B**), an increase in ALP expression (a and b), Col1 (c and d), phosphoserine containing proteins (P-serine) (e and f) were observed.

### TRIP-1 Overexpression Stimulates the Expression of TGF-β Signaling Modulators

**FIGURE 7:**
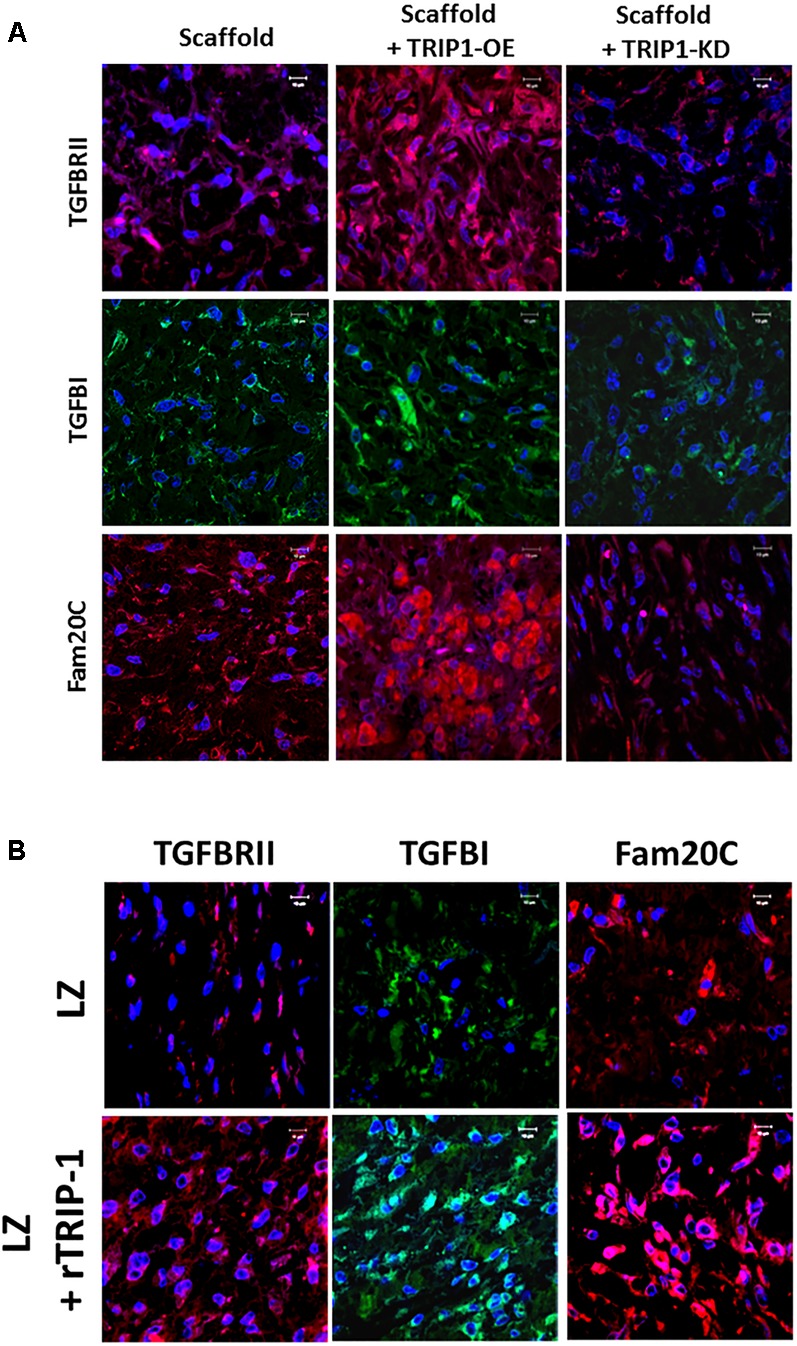
TRIP-1 and its effect on TGF-β signaling members and the secreted kinase of the ECM *in vivo*. **(A)** Effect of TRIP1-OE and TRIP1-KD cells on TGF-β ligand, its receptor and Golgi kinase Fam20C *in vivo*: Representative confocal micrographs showing the expression of TGF-βRII, TGF-β, and Fam20C in implanted scaffold containing control MC3T3-E1, TRIP1-OE, and TRIP1-KD cells, respectively. Scale bar represents 20 μm for all images. **(B)** Effect of rTRIP-1 adsorbed on LZ-scaffold on TGF-β ligand, its receptor and Golgi kinase Fam20C *in vivo*: Representative confocal micrographs showing the expression of TGF-βRII, TGF-β1, and Fam20C. Note increased expression levels of these markers with rTRIP-1 treatment. Scale bar represents 20 μm for all images.

TRIP-1 is a TGF-β receptor II binding protein, therefore, we investigated if TRIP-1 had an effect on the expression levels of TGF-β ligand and type II serine/threonine kinase receptor (**Figure 7A**). Interestingly, TRIP1-OE cells showed abundant expression of type II receptor within the cells and the ligand was highly expressed within the ECM matrix and lower expression in TRIP1-KD cells (**Figure 7A**). As *p*-serine staining showed higher level of phosphoproteins in the matrix, we sought to examine the levels of Fam20C (initially designated as DMP4 in our initial study) a secretory pathway kinase that phosphorylates proteins in the extracellular matrices of bone and teeth. Interestingly, TRIP1 overexpression showed high levels of Fam20C expression and was downregulated in TRIP1-KD cells (**Figure 7A**). Similar expression pattern was observed with rTRIP1 on LZ scaffold containing MC3T3-E1 cells (**Figure 7B**).

### TRIP-1 Overexpression Promotes Angiogenesis *in vivo*

**FIGURE 8:**
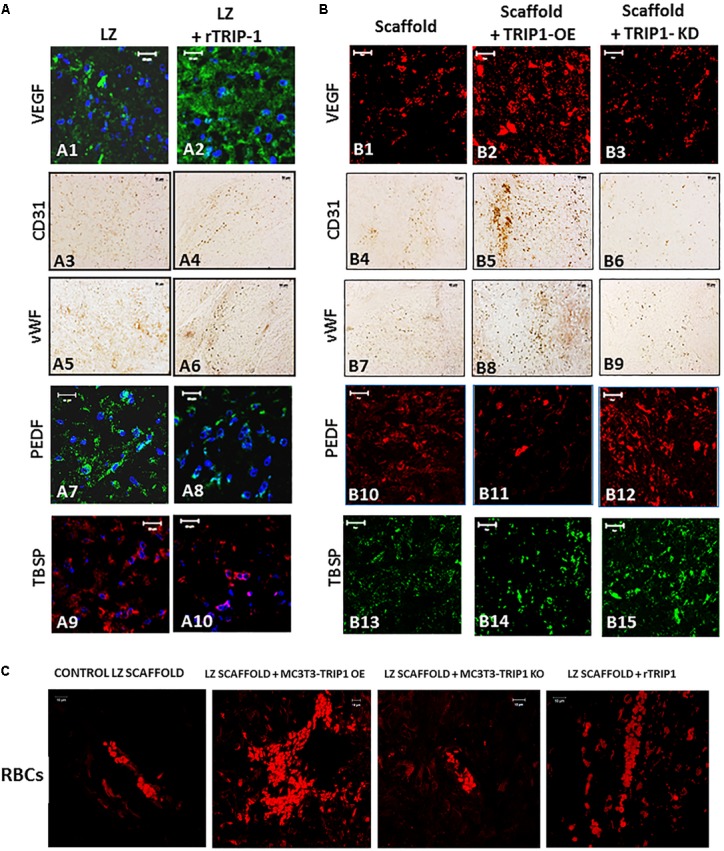
TRIP-1 promotes angiogenesis *in vivo*. **(A)** Effect of rTRIP-1 adsorbed on LZ-scaffold on angiogenesis: Representative confocal micrographs showing the immunohistochemical localization of VEGF **(A1,A2)**, CD31 **(A3,A4)**, vWF **(A5,A6)**, PEDF **(A7,A8)**, TBSP **(A9,A10)** in implanted control scaffold and scaffold +rTRIP-1, respectively. Note increased expression levels of angiogenesis and endothelial cell markers with rTRIP-1 treatment. Scale bar represents 20 μm for all images. **(B)** Effect of TRIP1-OE and TRIP1-KD cells on angiogenesis: Representative confocal micrographs showing the expression levels of VEGF **(B1–B3)**, CD31 **(B4–B6)**, vWF **(B7–B9)**, PEDF **(B10–B12)**, TBSP **(B13–B15)** in implanted scaffold containing control MC3T3-E1, TRIP1-OE, and TRIP1-KD cells, respectively. Scale bar represents 20 μm for all images. **(C)** TRIP-1 promotes the formation of red blood cells: Representative confocal images showing autofluorescence of RBCs in the different groups as stated.

We next explored if TRIP-1 modulated angiogenesis *in vivo*. Immunohistochemical analysis was performed with several pro-angiogenic and anti-angiogenic markers (**Figures 8A,B**). In the experimental group with LZ scaffold adsorbed with rTRIP-1, more staining for VEGF a pro-angiogenic marker was observed when compared with the control (A1–A2). VEGF was highly upregulated in TRIP1-OE cells when compared with the control and reduced expression in TRIP1-KD cells (B1–B3). VEGF staining pattern corroborated well with the number of RBCs in TRIP-OE cells and rTRIP-1 treated scaffolds (**Figure 8C**) implying TRIP1 promoted vascularization. Immunohistochemical analysis with endothelial cell markers CD31 and vWF were carried out to investigate if TRIP-1 aids vasculogenesis by recruiting endothelial cells. Results show CD31 was enhanced with TRIP-1 (A3–A4 and B4–B6). vWF showed a similar pattern of expression with TRIP1-OE cells and LZ-rTRIP-1 treated scaffolds when compared to control LZ containing MC3T3-E1 and MC3T3-KD cells (A5–A6 and B7–B9). Thus, the above results demonstrate that TRIP-1 influences angiogenesis *in vivo*.

Pigment epithelium-derived factor is an anti-angiogenic factor and decreased expression was observed both in recombinant TRIP-1 treated scaffolds and TRIP1-OE cells, however, expression was higher in the absence of TRIP1 (A7–A8 and B10–B12). Similar expression patterns were observed with thrombospondin (TBSP) an anti-angiogenic factor (A9–A10 and B13–B15).

## Discussion

The ECM of bone and dentin are unique, as it contains several matrix molecules which provide the structural framework to support cells and it also contains a rich milieu of cytokines and growth factors entrapped within the fibrillar matrix which conveys signals for various cellular functions (Posey et al., 2008; Alford et al., 2015). The signaling function from the ECM to the cells and from the cells to the ECM is a two-way dialog. Unique to bone matrix are several phosphoproteins, notably members of the SIBLING family that aid in hydroxyapatite nucleation and growth. Recently, we have identified TRIP-1 in the ECM of bone and dentin. This suggests that several proteins exist in the ECM that are synthesized by the osteoblasts and odontoblasts that might play a pivotal role in the organization of the mineralized matrices of bone and dentin.

In order to determine other potential functions of TRIP-1 in the ECM, preosteoblast MC3T3-E1 cells were genetically modified to overexpress or silence TRIP-1. Morphological examination by SEM showed that with TRIP-1 overexpression and in the presence of differentiation media the cells were elongated with several prominent cell processes and secreted large amounts of EV. SEM analysis further showed that the EVs were able to penetrate inside the dense fibrous ECM and they were specifically localized on the fibrillary strands. However, the mechanism by which the EVs are transported on the dense network of the ECM are yet to be studied. These EV appeared as functional component of the extracellular matrix. Published reports demonstrate that EVs include exosomes, matrix vesicles, and microvesicles, and carry a multi-molecular cargo of proteins, nucleic acids, lipids metabolites and signaling molecules (Rilla et al., 2017). TEM analysis show that the EV are enveloped by a lipid bilayer with an electron-dense core. Such nano-vesicular structures have been shown to carry calcium phosphate crystals and are transported to the ECM to initiate the process of mineral nucleation (Hasegawa et al., 2017). In cartilage, bone and dentin it has been shown that matrix vesicles also release their bioactive cargo to the ECM changing its properties, rather than to other cells (Chaudhary et al., 2016). TRIP-1 is one such protein that lacks a signal peptide, and we have earlier provided evidence that intracellular TRIP-1 can be packaged and exported to the ECM via exosomes. In this study, we demonstrate that TRIP-1 can be considered to play an important role in modifying the composition of the extracellular matrix by secreting EVs. Interestingly, the amount of EVs on the cell surface increased in the presence of TRIP-1. Knockdown of TRIP-1 attenuates EV deposition. Therefore, it is possible that TRIP-1 favors biogenesis of EVs and their secretion to the ECM to modulate mineralization and permit communication between different bone cell types, stem cells and endothelial cells in the microenvironment of the secreted vesicles.

ECM components in mineralized matrices not only function as a scaffold and supports mineralization but also influence structural flexibility, promote angiogenesis and other cellular functions to support structure and function. We have observed that overexpression of TRIP-1 and presence of immobilized TRIP-1 in the cellular microenvironment results in increased expression of key matrix proteins of mineralized tissues namely type I collagen and fibronectin. Collagen an important constituent of the connective tissue stroma in mineralized tissues play a vital role in maintaining structural integrity and function as a template in matrix mineralization. As collagen has a natural birefringence which is attributed to the arrangement of fibers and enhanced by picrosirius red staining, therefore, it is an ideal technique to visualize the structure of the *de-novo* collagen synthesized by the various cell types used in this study. Picrosirius red along with polarized light impart different birefringent colors in shades of green-yellow representing immature procollagen to orange-red depicting mature closely packed collagen fibers. Processing of these images with CT-FIRE fiber quantification software showed that TRIP-1 had an influence on the length and width of the collagen fibrils.

Besides providing a structural template for mineralization both fibronectin and collagen play a role in angiogenesis. Recently, it was observed that type I collagen was deposited on the endothelial wall of the blood vessels and served as a template for new bone during endochondral bone formation (Shoham et al., 2016). Specifically, a correlation between Col1A1 expression and vascular development was established in this study. The abundant deposition of fibronectin is interesting as it is required for matrix assembly and vascular morphogenesis. Polymerization of fibronectin is required for endothelial cell survival, proliferation and tube formation (Wijelath et al., 2004). Knockout mice of the splice variants of fibronectin EIIIA and EIIIB show severe vascular defects. Fibronectin-integrin binding is known to initiate a MAP-kinase dependent signaling cascade. Interestingly, TRIP-1 overexpression is able to induce the phosphorylation and activation of ERK1/2 and this would be an important signaling mechanism for cellular differentiation. Specificity of ERK1/2 activation was attenuated with TRIP1-KO cells.

Results show that TRIP-1 promotes higher expression of extracellular phosphoproteins as demonstrated by IHC analysis using phosphoserine antibody. Correspondingly, we have shown that Fam20C a kinase that catalyze the phosphorylation of secreted proteins is also upregulated with TRIP-1 overexpression. Fam20C has been shown to be a single kinase that generates the majority of the secreted phosphoproteome.

TGF-β cytokine can induce a diverse set of responses that is modulated by the cellular context. As TRIP-1 is a TGF-β receptor II interacting protein we investigated if it had an influence on the expression of type II receptor. Interestingly, TRIP-1 overexpression led to an increase in the expression of type-II receptor and higher TGF-β ligand localized in the ECM. It is well established that the TGF-β signaling is activated when the ligand binds to the type II receptor which then phosphorylates serine and threonine residues in the type I receptor, which subsequently propagates the signal through Smad activation (Wrana et al., 1994). The downstream targets of TRIP-1 mediated TGF-β signaling are yet to be identified. However, TGF-β can stimulate angiogenesis depending on the expression levels and the tissue context.

Vasculature in mineralized tissues play a significant role in the ossification process of bone and dentin. It is possible that TRIP-1 can activate TGF-β to promote the expression of a major regulator of angiogenesis VEGF (Yuan et al., 2014). Several studies have shown that matrix-bound VEGF induces prolonged activation of VEGFR2 leading to downstream signaling events (Sivaraj and Adams, 2016). Immobilized VEGF can also induce the association of cell surface integrin β-1 subunit with VEGFR2. Thus matrix-bound VEGF is important for bone angiogenesis. Interestingly, it has previously been demonstrated that vascular patterning directs bone formation (Shoham et al., 2016). Increase in expression of CD31 and vWF with TRIP-1 suggests the presence of endothelial cells in the ECM (Langen et al., 2017). CD31 a membrane glycoprotein is traditionally used as a marker for endothelial cells. It is possible that pre-osteoblasts might transdifferentiate into endothelial cells. In the bone marrow CD31^+^ cells are reported to be highly angiogenic and vasculogenic cells (Kim et al., 2010). von Willebrand factor is another glycoprotein produced by endothelial cells and is normally used as a marker to identify vessels and vessel density in tissue sections (Shi et al., 1998; Jazayeri et al., 2008; Wang et al., 2017). The presence of abundant RBCs in the scaffolds with TRIP-1 further suggest the ability of ECM from TRIP-1 overexpressing cells to favor formation of blood vessels.

TRIP-1 overexpression lowered the expression of PEDF and TSP (thrombospondin). PEDF, a member of the serine protease inhibitor (serpin) superfamily, has been described as one of the most potent anti-angiogenic factor in the ECM (Tombran-Tink et al., 2005; Chetty et al., 2015). PEDF is not only able to inhibit EC migration and proliferation but can also induce EC apoptosis (Broadhead et al., 2010). PEDF contains collagen-binding site and heparin-binding site, therefore, binding of ECM components can modulate its activity (Meyer et al., 2002; Sekiya et al., 2011). Thrombospondin is another matricellular extracellular matrix protein with an anti-angiogenic function during bone development.

Therefore, the EV from TRIP-1 overexpression might have a role in the transformation of the cells within its microenvironment to form endothelial cells. It is possible that TRIP-1 containing exosomes might contain the ingredients to promote vasculogenesis and angiogenesis.

## Conclusion

TRIP-1 is a recently identified ECM protein which has the ability to bind calcium and precipitate calcium phosphate polymorphs, therefore can work in concert with other matrix proteins to fine tune matrix mineralization. The mineralized matrix not only provides the mechanical strength to the tissue but contains several macromolecules embedded within the matrix that provide signaling cues and guides cell differentiation. In this study, we demonstrate that TRIP-1 promotes the secretion of EV and play an important role in modulating the composition of the matrix. Multiple macromolecules such as collagen and fibronectin which functions in the hierarchical organization of the ECM in mineralized tissues are influenced by the overexpression of TRIP-1. Transport of TRIP-1 to the ECM was shown to be facilitated by exosomes. TRIP-1 in the ECM can promote both direct and indirect effects on adhering preosteoblasts and consequently stimulate cell differentiation and angiogenesis. A better understanding of the multifunctional role of TRIP-1 in regulating osteogenesis and angiogenesis is essential for the development of therapeutics for mineralization-related disorders.

## Author Contributions

YC performed the experiments and assembled the data. AG analyzed the data and wrote the manuscript.

## Conflict of Interest Statement

The authors declare that the research was conducted in the absence of any commercial or financial relationships that could be construed as a potential conflict of interest.
